# Glutamate synthases from conifers: gene structure and phylogenetic studies

**DOI:** 10.1186/s12864-018-4454-y

**Published:** 2018-01-19

**Authors:** Ángel García-Gutiérrez, Francisco M. Cánovas, Concepción Ávila

**Affiliations:** Departamento de Biología Molecular y Bioquímica, Facultad de Ciencias, Universidad de Málaga, Campus de Teatinos, 29071 Málaga, Spain

**Keywords:** BAC, Gene structure, Intron-exon boundaries, Megagenomes, Phylogenie

## Abstract

**Background:**

Plants synthesize glutamate from ammonium by the combined activity of the enzymes glutamine synthetase (GS) and glutamate synthase (GOGAT) through the glutamate synthase cycle. In plants, there are two forms of glutamate synthases that differ in their electron donors, NADH-GOGAT (EC 1.4.1.14) and Fd-GOGAT (EC 1.4.7.1), which have differential roles either in primary ammonia assimilation or in the reassimilation of ammonium from different catabolic processes. Glutamate synthases are complex iron-sulfur flavoproteins containing functional domains involved in the control and coordination of their catalytic activities in annual plants. In conifers, partial cDNA sequences for GOGATs have been isolated and used for gene expression studies. However, knowledge of the gene structure and of phylogenetic relationships with other plant enzymes is quite scant.

**Results:**

Technological advances in conifer megagenomes sequencing have made it possible to obtain full-length cDNA sequences encoding Fd- and NADH-GOGAT from maritime pine, as well as BAC clones containing sequences for NADH-GOGAT and Fd-GOGAT genes. In the current study, we studied the genomic organization of pine GOGAT genes, the size of their exons/introns, copy numbers in the pine genome and relationships with other plant genes. Phylogenetic analysis was performed, and the degree of preservation and dissimilarity of key domains for the catalytic activities of these enzymes in different taxa were determined.

**Conclusions:**

Fd- and NADH-GOGAT are encoded by single-copy genes in the maritime pine genome. The *Fd-GOGAT* gene is extremely large spanning more than 330 kb and the presence of very long introns highlights the important contribution of LTR retrotransposons to the gene size in conifers. In contrast, the structure of the *NADH-GOGAT* gene is similar to the orthologous genes in angiosperms. Our phylogenetic analysis indicates that these two genes had different origins during plant evolution. The results provide new insights into the structure and molecular evolution of these essential genes.

**Electronic supplementary material:**

The online version of this article (doi: 10.1186/s12864-018-4454-y) contains supplementary material, which is available to authorized users.

## Background

Nitrogen assimilation is an extremely important physiological process for plant growth and development. Inorganic nitrogen is assimilated into the amino acids L-glutamine and L-glutamate through the glutamine synthetase/glutamate synthase (GS/GOGAT) cycle. Glutamine synthetase (GS) catalyzes the ATP-dependent incorporation of ammonium (NH_4_^+^) to glutamate to produce glutamine. Glutamate synthase (GOGAT) catalyzes the conversion of L-glutamine and 2-oxoglutarate into two molecules of L-glutamate, one of which participates in further ammonium assimilation via GS, and the other of which is used as a nitrogen donor for the production of all nitrogen-containing molecules [1].

Plants contain two types of GS, GS1 and GS2, localized in the cytosol and chloroplast, respectively. The composition, gene number and expression level of the GS gene family have been thoroughly studied in plants. Phylogenetic studies of nucleotide and amino acid composition have shown that genes for chloroplastic and cytosolic GS in plants come from a common ancestor that diverged before the division between angiosperms and gymnosperms [2]. The molecular analysis of GS isoenzymes in different plant species has revealed their specialization and non-overlapping roles [3, 4].

Two molecular forms of glutamate synthase that differ in their electron donor for catalysis, NADH-GOGAT (EC 1.4.1.1) and ferredoxin (Fd)-GOGAT (EC 1.4.7.1.), are present in plants. Both isoforms are located in the plastids [5] and generally differ in terms of molecular mass, kinetics, tissue distribution and function in plant nitrogen metabolism. The biological functions of Fd-GOGAT and NADH-GOGAT are tightly related to the regulation by light and metabolite sensing-systems [6]. Fd-GOGAT is the major isoenzyme in leaves, accounting for 95% of the activity in *Arabidopsis*, while NADH-GOGAT is a minor enzyme represented in leaves [7]. The high levels of Fd-GOGAT in leaves are consistent with a major role of the enzyme in N primary assimilation and in photorespiration [8, 9]. Biochemical studies of NADH- GOGAT have shown that it is primarily located in plastids of non-photosynthetic tissues such as roots, where it is involved in N primary assimilation and N reassimilation from catabolic processes [9].

DNA sequences for cDNA and genes have been characterized for Fd- and NADH-GOGAT from several plant species. The gene family is represented by a small number of members. Fd-GOGAT is encoded by one or two genes in most angiosperm species; for example, in *Arabidopsis,* two Fd-GOGAT genes, *GLU1* and *GLU2* have been reported [10]. The Fd-dependent plant protein is monomeric and contains a transit peptide for its localization in the chloroplast. The complete sequence of Fd-GOGAT in angiosperms is related to the sequence of *gltB* gene that encodes the α subunit of the (αβ) hexameric bacterial NAD (P)H-glutamate synthase. However, no similarities have been reported between the plants Fd-glutamate synthase genes and the *gltD* gene encoding the β subunit of the bacterial NADPH-glutamate synthase [11].

A small gene family, *GLT,* consisting of one or two members, encodes plant NADH-glutamate synthases. The *GLT* gene contains conserved sequences for *gltB* and *gltD* found in prokaryotic NAD (P)H-glutamate synthases. The plant NADH-GOGAT polypeptide is also monomeric, similar to the Fd-GOGAT polypeptide, but contains a β subunit–like polypeptide fused at the C-terminus of the α subunit –like polypeptide.

The study of the molecular biology of ammonium assimilation in gymnosperms has revealed the existence of two different modes of regulation of ammonium assimilation in photosynthetic tissues [12]. In species of conifers showing light-independent chloroplast development, the biosynthesis of glutamine and glutamate is compartmentalized between the cytosol and the chloroplast [13]. To account for nitrogen assimilation in green pine tissues, the presence of two isoforms has been previously reported: a highly expressed Fd-GOGAT isoform that, together with GS1a, initiate the GS/GOGAT cycle [12, 14] and an NADH-GOGAT isoform that is present in low amounts in green pine tissues [15]. Subsequent expression analysis performed in our laboratory using pine seedlings and one-year-old pine trees showed that the *NADH-GOGAT* gene is expressed predominantly in non-photosynthetic tissues such as roots and stems (Additional file 1: Figure S1). This finding suggests a role of NADH-GOGAT in the biosynthesis of glutamate in vascular tissues associated with the recycling of ammonium released in lignifying cells [16, 17].

In regard to GOGAT genes of conifers, a partial sequence of cDNA encoding Fd-GOGAT has been previously reported and used for expression studies in maritime pine [15]. Following those studies, in the European project Sustainpine, the *P. pinaster* transcriptome was assembled [18], and the full-length c-DNA sequences of Fd-GOGAT and NADH-GOGAT from this species were obtained.

Little is known about the genomic organization of these genes in conifers because the large sizes of the genomes have hampered their de novo sequencing [19]. Consequently, the drafts of only a few conifer genomes are available [20–22]. With this prospect, the analysis of BAC clones has been a common approach used to target of gene-rich regions in conifer species. Two BAC clones containing part of the Fd-GOGAT gene encoding the C-terminus of the protein in maritime pine were characterized [23]. However, to date, no partial or complete sequence of a conifer NADH-GOGAT gene is available.

In the present work, by screening a maritime pine BAC library, a BAC clone containing the full-length sequence of the *NADH-GOGAT* gene from *P. pinaster* was isolated. In parallel, the full genomic structure of the *Fd-GOGAT* gene was retrieved from the *P. taeda* genome [22]. A comparison was made between the *P. pinaster* BAC clone containing a partial sequence of the maritime pine gene [23] and the scaffolds containing the full genomic structure of the *Fd-GOGAT* gene from *P. taeda,* which was available through the Dendrome project. The structures of the two pine GOGAT genes were analyzed in depth, and sequences were used to perform phylogenetic and comparative studies with other organisms to understand the molecular evolution of these genes in plants.

## Results

### *Fd-GOGAT* and *NADH-GOGAT* gene structure in pine

**Fig. 1:**
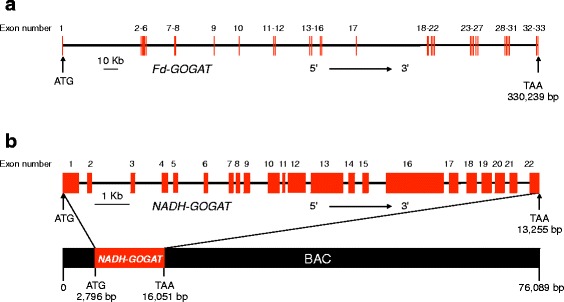
Structure of the *Fd-GOGAT* and *NADH-GOGAT* genes in pine. **a** *Fd-GOGAT* gene structure. Light red lines represent exons, and the intervening lines represent introns; the exon number is indicated. The scale bar represents 10 kb. **b** *NADH-GOGAT* gene structure contained in the BAC clone, GenBank (KP172184). Light red boxes represent exons, and the intervening lines represent introns. Exon numbers are also indicated. The scale bar represents 1 kb. The position of the gene in the BAC clone is highlighted

To determine the GOGAT gene structures in pine, we followed two different strategies based on the available information for both genes. In an earlier work from our group [23], a partial genomic sequence for maritime pine *Fd-GOGAT* that encodes the carboxy-terminal region of the protein was obtained. Using this genomic information together with the cDNA full-length sequence derived from the maritime pine transcriptome [18], we searched the *P. taeda* scaffolds from the Dendrome database and obtained scaffold number 377118 containing the *Fd-GOGAT* gene sequence that was retrieved from the NCBI database (Fig. 1a).

The gene is 330,239 bp long and encodes a protein of 1630 amino acids. The coding sequence is organized into 33 exons separated by 32 introns, some of which have sequences as long as 50 kb (Additional files 2 and 3: Tables S1 and S2). This exon/intron structure is well conserved in other plants such as *Populus trichocarpa, Arabidopsis thaliana* and *Oryza sativa* (Additional file 4: Figure S2A). However, the intron size for these genes in angiosperms is much smaller, and the gene structure spreads across regions of the genome between 8704 bp for the *Arabidopsis* gene and 16,033 bp for the poplar gene. In the moss *Physcomitrella patens,* the gene contains 29 exons and 11,049 bp, and in the green algae *Chlamydomonas reinhardtii,* the gene contains 11 exons and 7719 bp (Additional file 4: Figure S2A).

To obtain the *NADH-GOGAT* gene structure, a BAC library from maritime pine was screened in this work. Following the procedure described in Materials and Methods, a BAC clone hybridizing to the NADH-GOGAT probe was isolated. The BAC clone contained a 76 kb insert including the whole *NADH-GOGAT* gene structure. The gene spans a region of 13.2 kb and, as in other plants, encodes a single polypeptide of 2218 amino acids (Fig. 1b). In the BAC insert, there were no other sequences encoding genes. The sequence assembly was deposited into the GenBank database (KP172184). The gene is organized into 22 exons with introns no longer than 1 kb (with the exception of I2) (Additional files 3 and 5: Tables S2 and S3) (Fig. 1b). These results are similar to those found in other plants such as *P. trichocarpa* or *O. sativa* where the gene is also organized into 22 exons (Additional file 4: Figure S2B)*.* However, the gene in *A. thaliana* has 20 exons. In all plant species, including pine, the gene spans regions with similar sizes between 10 and 13 kb and has similar intron lengths. Similar to the *Fd-GOGAT* gene structure, the *NADH-GOGAT* gene structure is not conserved in the moss *P. patens,* where the gene contains 25 exons. However, a similar number of exons (22) was identified in the green algae *C. reinhardtii*.

### Contribution of transposable elements to the structures of the *Fd-* and *NADH-GOGAT* genes

**Fig. 2:**
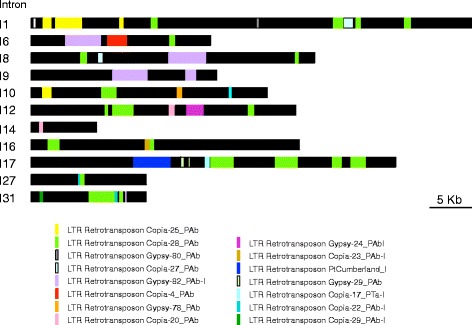
Distribution of retrotransposons present in the eleven introns bigger than 11 kb in the pine *Fd-GOGAT* gene. Different *Copia* and *Gypsy* LTR retroelement families are represented with different colors

The large conifer genome size is in part due to transposon activity, especially LTR retrotransposons that are major players in genome size variation. Taking this situation into account, a detailed study was conducted to detect the presence of these elements in the pine *GOGAT* genes. The scaffold of the *P. taeda* assembly containing the *Fd-GOGAT* was searched for transposons in the unusually large introns of the gene. This study included the 11 longest introns (Additional file 3: Table S2), present in the gene sequence (Fig. 2). Most of the selected introns consisted of long sequence stretches containing multiple LTR retrotransposons, except I14, the smallest of the long introns included in the study. The most commonly found LTR retrotransposons belonged to the families *Copia* and *Gypsy*, with copies of Gypsy-82_PAb-I filling regions in introns over 5 kb in: I6, I8 and I9; Copia-28_PAb was also a well-represented LTR retrotransposon in introns I1, I6, I8, I10, I12, I16, I17, I27 and I31.

**Fig. 3:**
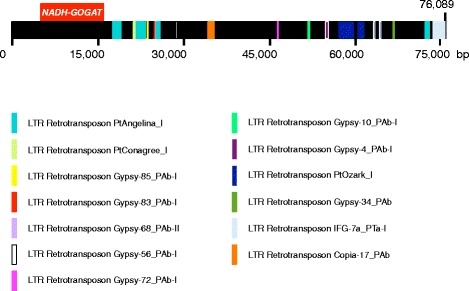
LTR retrotransposons located in the intergenic region of the *NADH-GOGAT* gene in the BAC clone. Different classes of LTR retrotransposons are marked in different colors. The red box represents the NADH-GOGAT gene

A similar study was performed with the BAC sequence containing the *NADH-GOGAT* gene (Fig. 3). The analysis revealed that the retrotransposons present in the BAC clone were located outside the *NADH-GOGAT* gene sequence, in the intergenic region. No long stretches of LTR elements were found inside the gene, since it is a compact gene structure with small introns. The *Gypsy* retrotransposon family was the most represented. However, the *Gypsy* repetitions found in the *NADH-GOGAT* BAC clone belong to a different class than those described for the *Fd-GOGAT* gene structure. The distribution of retrotransposons in this case did not present long regions containing the same retrotransposon over thousands of bases as in the *Fd-GOGAT* gene.

### Estimation of *Fd- and NADH-GOGAT* gene copies in the maritime pine genome

**Fig. 4:**
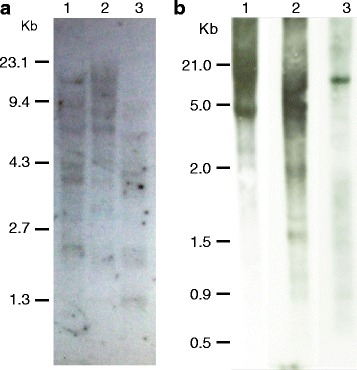
Southern blot analysis to determine gene copy number. **a** A Southern hybridization was performed on 10 μg of *P. pinaster* DNA digested with three restriction enzymes. Lane 1: *BamHI*, Lane 2: *SalI* and Lane 3: *HindIII* were hybridized to a ^32^P–labelled probe of the *Fd-GOGAT* gene. **b** Southern blot analysis to determine the copy number of the *NADH-GOGAT* gene in pine. In total, 10 μg of *P. pinaster* DNA was digested with three restriction enzymes. Lane 1: *BamHI*, Lane 2: *SalI* and Lane 3: *HindIII* were hybridized to a ^32^P–labelled probe of the *NADH-GOGAT* gene. Numbers on the left correspond to DNA molecular markers in kb

Pine genomic DNA isolated from maritime pine was digested with three different restriction enzymes, *BamHI*, *SalI* and *HindIII;* electrophoresed and transferred to a membrane for Southern blot analysis (Fig. 4). The use of a specific Fd-GOGAT probe (Fig. 4a) displayed multiple bands in each lane, suggesting the presence of more than one gene in the genome or, alternatively, that the sequence used as the probe matched a genomic sequence containing more than one restriction site for the enzyme used. However, a single *Fd-GOGAT* transcript was identified in the maritime pine transcriptome using a RNA-seq approach. When the same analysis was performed using an NADH-GOGAT probe (Fig. 4b), a single band of approximately 13 kb, the gene size, was observed in the *SalI* lane. Furthermore, a single *NADH-GOGAT* transcript was identified using the RNA-seq approach. These experimental results suggest that a single gene encoding NADH-GOGAT is present in the pine genome.

### Fd-GOGAT and NADH-GOGAT protein functional domains

**Fig. 5:**
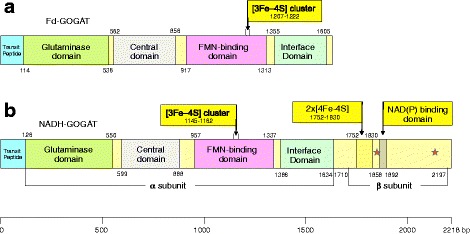
Functional domains of the pine GOGAT proteins. **a** Modular protein structure of the Fd-GOGAT pine protein. The starting and final amino acids of each domain are indicated. **b** Modular protein structure of the NADH-GOGAT pine protein. The starting and final amino acids of each module are indicated. The analogous sequence of the α and β subunits are indicated in the pine protein. The red stars indicate the consensus sequences for the binding of the adenylated portion of FAD. Modules with the same function in both proteins are represented with the same color

The pine ferredoxin-dependent glutamate synthase ((Fd-GOGAT) is formed by a single polypeptide similar in size (1630 amino acids), to the α subunit of the bacterial NADPH-GOGAT. However, NADH-glutamate synthase (NADH-GOGAT) is formed by a long single polypeptide, 2218 amino acids long, corresponding to the fusion of the α and β bacterial subunits. A comparison of the protein domain distribution has been performed for the two GOGATs from pine (Fig. 5). Both proteins possess a transit peptide in the amino-terminal region for its plastidial localization of 113 amino acids for Fd-GOGAT and 125 amino acids for NADH-GOGAT. Additionally, both proteins contain a glutamine amido-transferase class II or glutaminase domain 423 or 424 amino acids long in the N-terminal region. The glutaminase domain encodes a class II purF-type glutamine amidotransferase region, where glutamine is hydrolyzed, releasing ammonium. Next, the central domain is located between residues (562–856) for Fd-GOGAT and (599–888) for NADH-GOGAT. This domain is part of the structure that gives rise a tunnel to transfer the ammonia produced in the hydrolysis of the glutamine to the synthase site of the enzyme to continue the catalytic reaction.

The synthase domain contains (a) the FMN cofactor, amino acid residues (917–1313) in Fd-GOGAT and (957–1337) in NADH-GOGAT and (b) the [3Fe-4S] cluster on the surface of both proteins to receive the electron transferred from the corresponding cofactors. In NADH-GOGAT, the NADH-binding domain is positioned in the C-terminal region of the protein that corresponds to the bacterial β subunit, amino acid residues (1858–1892). In NADH-GOGAT, there are also two [4Fe-4S] clusters located in the C-terminal region of the protein (residues 1752–1830). A molecular model of the bacterial protein has clarified the electron transfer pathway from the FAD on the β subunit, to the FMN on the α subunit, through the low potential [4Fe-4S] centers on the β subunit and the [3Fe-4S] cluster in the α subunit [24]. There is an interface-like domain in the C-terminus of Fd-GOGAT (amino acid residues 1355–1605) or in the linking region between the α and β prokaryotic subunit-like (1386–1634) of NADH-GOGAT. This interface-like domain is supposed to maintain the structural requirements of the proteins but is not involved in catalysis.

### Evolutionary relationships of GOGAT proteins in different lineages

**Fig. 6:**
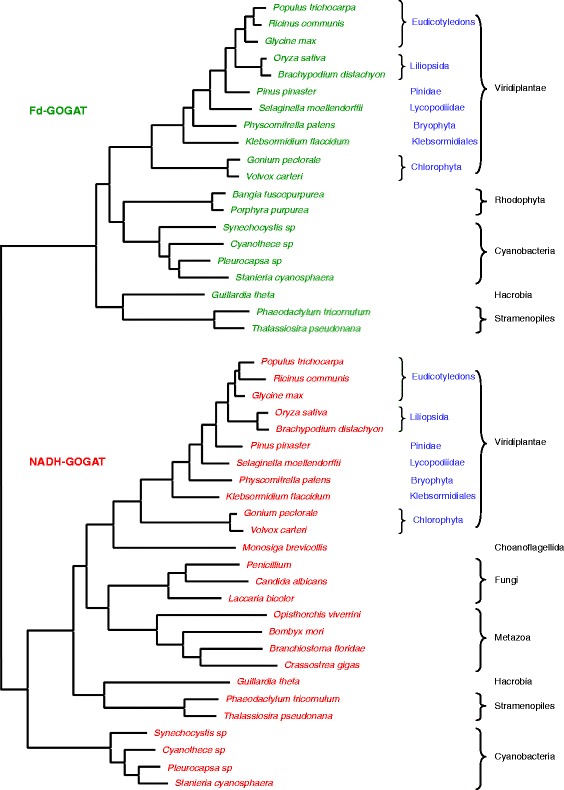
Phylogenetic tree of Fd-GOGAT and NADH-GOGAT genes. The phylogenetic reconstruction was made by the maximum likelihood statistical method, and the phylogeny was tested with 50 bootstrap replications. The evolutionary distances were computed using the JTT matrix-based method, and the rates among sites were uniform. All these analyses were conducted in MEGA6

With the availability of new sequencing information from different plant species including conifers, we analyzed the evolutionary relationships between the two GOGAT types. Selected Fd- and NADH-GOGAT protein sequences from representative eukaryotic species and the cyanobacteria species were phylogenetically compared (Fig. 6). The same species were used for the comparative study of both GOGAT proteins, except Fungi and Metazoa that lack Fd-GOGAT and the Rhodophyta that lack NADH-GOGAT. The resulting tree classified Fd-GOGAT and NADH-GOGAT sequences into two separated groups. The first ramification of Viridiplantae was subdivided in eudicotyledons and monocotyledons or Liliopsida, and the following taxonomic ranks of Viridiplantae were present in ramifications increasingly distant based on the evolutionary distance: Pinidae, Lycopodiidae, Bryophyta, Klebsormidiales and finally Chlorophyta. Interestingly, in the Fd-GOGAT group, the evolutionary branch closest to Viridiplantae contained the Rhodophyta algae and the prokaryotic cyanobacteria, whereas the farthest branch grouped the eukaryotic phylum Hacrobia and Stramenopiles, both considered Chromalveolates. In contrast, in the NADH-GOGAT group, the closest phylum to the green plants was the Choanoflagellida, and a second, more remote branch was subdivided in Fungi and Metazoa. The next branch contained again together Hacrobia and Stramenopiles, and the last ramification was the prokaryotic cyanobacteria.

The Fd- and NADH-GOGAT structures of different organisms were further compared by proportionally representing the sizes of the proteins and considering the presence or absence of subunits (Additional file 6: Figure S3). The species included in the comparison were eukaryotes; the reference that was used was the prokaryotic *Synechocystis*, which has both Fd- and NADPH-GOGAT proteins, and the proteobacteria *Azospirillum*, which only contains NADPH-GOGAT. All Viridiplantae species had Fd-GOGAT and NADH-GOGAT sequences. The Spermatophyta sequences came from *Populus,* a eudicotyledon, and *Oryza*, a Liliopsida. The other Viridiplantae taxonomic ranks included were Pinidae (*Pinus*), Bryophyt*a* (*Physcomitrella*), Klebsormidiales (*Klebsormidium*) and Chlorophyta (*Volvox*). Additionally, the phylum Cryptophyt*a* (*Guillardia*) was incorporated in this study. The Opisthokonta, which only has NADH-GOGAT, was represented with the Metazoa *Bombyx* and the fungus *Laccaria*. The protein structure of the Fd-GOGAT proteins in all phyla considered corresponded to a single subunit whose size is very similar for most of the phyla except for the Rhodophyta *Porphyra* and the cyanobacteria *Synechocystis,* which exhibits smaller sizes (50–100 amino acid residues shorter than the other Fd-GOGATs).

NADPH-GOGAT proteins in most bacteria are integrated into two different subunits (large α and a small β), but in plants, they are monomeric, with a single polypeptide corresponding to the fusion of the bacterial α and β subunits, and are encoded by a single gene [25]. The resulting fusion in most cases yields a polypeptide between 50 and 250 amino acids longer than the sum of the prokaryotic α and β polypeptides.

## Discussion

### The structure of *GOGAT* genes in pine

In this work, the structures of the *Fd-GOGAT* and *NADH-GOGAT* genes in pine were analyzed. The sequence for *NADH-GOGAT* was obtained from a *P. pinaster* BAC clone while the gene structure for *Fd-GOGAT* was reconstructed by a comparative analysis between the *P. taeda* scaffolds, the partial genomic sequence and the full-length cDNA from *P. pinaster.*

The *Fd-GOGAT* gene is extremely large, expanding 330,239 bp and it is organized into 33 exons separated by 32 introns encoding a protein of 1630 amino acids. The positive relationship that seems to exist between the genome size and intron length [26] is particularly true for the *Fd-GOGAT* gene since the first intron is longer than 50 kb and 11 introns are greater than 13 kb (Fig. 1a; Additional file 3: Table S2). This structure is consistent with the multiple bands observed in the Southern blot of Fd-GOGAT because the probe identified many genomic fragments as the consequence of multiple restriction sites in the large introns (Fig. 4). Although a single Fd-GOGAT transcript has been identified in the maritime pine transcriptome, the presence of pseudogenes or truncated copies in the pine genome cannot be ruled out.

In the literature, it has been described that the length of introns can be related to gene function and to the level of expression [27]. In fact, previous reports in plants, unlike animals, claim that highly expressed genes tend to be less compact than low expressed genes and that the number and intron size correlate positively with highly expressed genes [28], which could be the case for pine *Fd-GOGAT*. The finding of a first long intron in the pine gene would be consistent with a significant trend of increased length observed in the first introns across the eukaryotic genes [29].

The presence of short sequences mediating the positive effect of introns on gene expression has also been described. These sequences have been termed intron-mediated enhancement (IME), and they have a general effect in eukaryotic genes from vertebrates, invertebrates, fungi and plants [30]. We searched for the presence of IME signals that could act as enhancers of the expression along the first intron of the *Fd-GOGAT* gene, and we have found many of these short sequences in the long 50 kb first intron of the *Fd-GOGAT* gene. It has also been reported that their function is probably involved in making the transcription machinery more processive to obtain full-length polyadenylated mRNAs.

In contrast, the features described above for the *Fd-GOGAT* gene are not present in the structure of the *NADH-GOGAT* gene. The intron sizes are not as long, oscillating between 1103 bp for the second intron I2 and 83 bp for the I8 (Fig. 1b). However, the intron number of the *NADH-GOGAT* gene is greater than the average of most genes. We also searched for IME sequences in the first two introns of the gene, those closest to the 5′ end. We found some of these IME signals in I2 but none in I1, and this could be explained by the small size of the first intron. Nevertheless, the possibility that the IME sequences in the second intron of the *NADH-GOGAT* gene may have an effect on gene expression cannot be ruled out.

### The presence of transposable elements in the pine *GOGAT* genes contributes substantially to the gene size

A common characteristic of conifer genomes is the presence of highly repetitive non-coding sequences [21, 31]. The presence of retrotransposons in the intronic sequences may also contribute to the increase in genome size of conifer species. Moreover, is quite common for these long repeats to be part of the introns detected in woody plants [32] unlike other plant species, where these long repeats are located in the intergenic regions. It has been suggested that this characteristic of conifer genes may be due at least in part to low DNA removal rates [21]. This situation appears to be the case for the *Fd-GOGAT* gene, where the size expansion is mainly due to the repeat insertions of LTR retrotransposons in 13 unusually long introns (Fig. 2). The *Copia* and *Gypsy* superfamilies are the most abundantly represented, with Gypsy-82_PAb-I and Copia-28_PAb covering stretches longer than 5 kb in some of the introns. Most of these transposable elements have been described as ancient transposable subfamilies that are present in almost all conifer genera [21]. However, further genomic studies are necessary to determine whether species-specific expansions of transposable elements families have occurred in *P. pinaster* as previously described in *Picea abies* [22] and *Picea glauca* [26]. In any case, the long stretches containing retrotransposons contributed substantially to increase the size of the pine *Fd-GOGAT* gene. This feature, which is decisive in determining the Fd-GOGAT final gene size, is not observed in the pine NADH-GOGAT gene that shows a similar structure to that of angiosperm genes. The search for retrotransposons in the sequence of the *NADH-GOGAT* BAC clone allowed the identification of long repeats exclusively placed in the intergenic region, which is similar to their location in most genes of angiosperms (Fig. 3).

### Functional domains are conserved in the pine GOGAT proteins

Glutamate synthases are complex iron-sulfur flavoproteins in which different protein segments are involved in the control and coordination of the partial catalytic activities of these enzymes. The structure of these complex proteins is well conserved in different plant species [33], and pine is not an exception (Fig. 4). The GOGAT proteins exhibit a modular architecture with a common region responsible for the glutamine-dependent glutamate synthesis from 2-oxoglutarate. A purF-type amidotransferase domain in the amino-terminal region of the protein is coupled to the synthase domain. Both modules are interconnected through a tunnel for the ammonium transfer similar to the GOGAT proteins from other species [34]. The main difference between the two proteins is the binding domain for the electron donor, to attach ferredoxin in the Fd-GOGAT and the pyridine nucleotide-binding domain in the NADH-GOGAT protein.

### Molecular evolution of GOGAT proteins

The phylogeny of the GOGAT proteins is a puzzling task since the original connections between eukaryotic linages are basically unknown, and the endosymbionts that originated the primitive mitochondria and plastid have not yet been characterized.

Fd-GOGAT is present in all photosynthetic organisms. The eukaryotic photosynthetic cells were originated by a primary endosymbiotic event [35] in which a free-living cyanobacteria-like prokaryote containing an Fd-GOGAT gene was engulfed by phagocytosis by a eukaryotic acceptor [36], generating the ancestor of three different supergroups also known as Archaeplastida: red algae, glaucophytes and green plants (Viridiplantae) [37]. The phylogenetic tree of Fd-GOGATs is consistent with the above hypothesis with Viridiplantae proteins clustered in a separate clade from Rhodophyta and cyanobacteria (Fig. 6).

An early step in primary endosymbiosis was the controlled exchange of metabolites between partners that was established in the ancestor of Chlorophyta and Rhodophyta*,* further supporting a monophyletic origin of photosynthetic organisms included in Archaeplastida [38]. The close position in the tree of Fd-GOGAT proteins between the cyanobacteria *Stanieria* and the Rhodophyta *Porphyra purpurea* suggests conservation of the primitive sequence in the red algae. Whereas the *Fd-GOGAT* gene of green plants is located in the nuclear genome, extant red algae still maintain the gene in the chloroplast genome [39]. The hypothesis of a common origin for Fd-GOGAT in green plants is reinforced, considering that an eventual primitive endosymbiotic gene transfer from cyanobacteria to the nucleus of the green algae occurred after the divergence between green and red algae from their common mitochondrion-bearing ancestor [40, 41]. Furthermore, the proximity of the phyla Hacrobia and Stramenopiles supports that the endosymbiotic gene transfer to the nuclear genome of the *Chromalveolata* was an early event [42, 43].

What is the origin of the GOGAT genes? The genome of the archaea *Methanococcus jannaschii* contains an open reading frame encoding a putative GOGAT one third the length of the large subunit [44]. Moreover, the genomes of several archaeal species of the genus *Pyrococcus* harbor sequences with homology to the small subunit of GOGAT genes in bacteria [45]. The occurrence of large and small subunit homologs in archaea suggest that GOGAT genes, as many others in bacteria, may have originated by horizontal gene transfer from archaea [45, 46].

What is the origin of the Fd-GOGAT gene? Most bacteria have NADPH-GOGAT consisting of a large subunit and small subunit. However, Fd-GOGAT is specific to photosynthetic organisms such as cyanobacteria and plants and directly receives reduction equivalents from ferredoxin, the final electronic acceptor of the photosystem I, a role that is fulfilled by the small subunit of the NADPH-GOGAT enzyme. Considering that most bacteria have NADPH-GOGAT, and that only the cyanobacteria have Fd-GOGAT, a plausible hypothesis would be that Fd-GOGAT originates from a duplication of the large subunit of a primitive cyanobacterial GOGAT. Both proteins have similar sequences, share almost identical domains, and catalyze the same reaction [34, 47]. The absence of NADH-GOGAT in the red algae *Porphyra* would not support the above hypothesis since Rhodophyta diverged from the same ancestor as green algae. However, the massive loss of genes in the red algae ancestor is a well-documented phenomenon [48]. However, the occurrence of Fd-GOGAT in photosynthetic chromalveolates is consistent with the above data because these organisms evolved by secondary endosymbiosis through the capture of a red algae cell by a primitive heterotrophic eukaryote [49].

NADH-GOGAT in eukaryotes is a very large monomeric protein (Fig. 5) likely resulting from the fusion of the genes encoding the prokaryotic α and β subunits [50]. Consequently, the gene had to be present in the eukaryotic ancestor and originated by endosymbiosis of an alpha proteobacteria (candidate for mitochondria) with a primitive eukaryotic, bacterial or archaeal host [51]. The presence of the NADH-GOGAT gene in animals and fungi suggest an early origin during the evolution of eukaryotes before the separation of photosynthetic and nonphotosynthetic organisms (Fig. 6).

## Conclusions

In this work, we established the gene structures of the *GOGAT* genes in pine. Single genes organized in 33 and 22 exons respectively encode the Fd- and NADH-GOGAT proteins. The presence of very long introns in the *Fd-GOGAT* gene highlights the important contribution of LTR retrotransposons to the gene size of conifer genes. We also found short sequences contributing to intron-mediated enhancement (IME) in the first introns of both genes that may play a putative role in their transcription. The modular protein structure of these complex proteins is well conserved in the pine representatives. Our phylogenetic analysis indicates that these two genes had different origins during plant evolution. The monomeric NADH-GOGAT of plants was already present in the eukaryotic host, while the Fd-GOGAT was supplied by the cyanobacteria-like endosymbiont.

## Methods

### Obtaining a BAC clone containing the *NADH-GOGAT* gene from maritime pine

The isolation of a BAC clone containing the *NADH-GOGAT* gene was performed as described previously [52]. The probe used was derived from the cDNA of maritime pine NADH-GOGAT, GenBank accession number: KY215940. A maritime pine pooled BAC library (0.8 × coverage) was screened*.* The pooled library had 83 bacterial stocks containing 4000 distinct clones each. Primary screening was performed by PCR, and putative positive PCR product pools were sequenced on a CEQ 8000 automated capillary sequencer (Beckman Coulter, Barcelona Spain). Next, the positive original cell pools were individualized in 36 × 384-well plates using a QPIX2 (Genetix).

Secondary screening was performed in high-density 22.2 × 22.2 cm nylon membranes hybridized to a [^32^P]-labeled specific genomic probe of 808 bp containing an intron sequence of 319 bp in the *NADH-GOGAT* gene. This probe was obtained from the amplification of genomic DNA using specific primers versus a partial 486 bp cDNA sequence.

### Bioinformatic methods

The sequences obtained using the FLX-454 system were processed by the UMA Bioinformatics Platform using two tools: SeqTrimNext pipeline (http://scbi.uma.es/seqtrimnext) [53] for sequence preprocessing and MIRA 3 [54] for assembly.

The scaffold containing the *P. taeda Fd-GOGAT* gene sequence was obtained from NCBI (http://www.ncbi.nlm.nih.gov/) according to the accession number (377118) provided by the Dendrome portal. The *Pinus taeda* Fd-GOGAT CDS sequence was obtained from Dendrome (https://treegenesdb.org/Drupal). The *P. pinaster* c-DNA sequences were obtained from the Sustainpine database information combined with RNA-seq data. The GenBank accession number was KY215941.

Retrotransposons in the genomic sequences were located using the default values of the CENSOR software tool [55] provided in the GIRI portal of the Genetic Information Research Institute (http://www.girinst.org/).

Searching for regions with similarity between sequences, the NCBI BLAST program (The Basic Local Alignment Search Tool: http://blast.ncbi.nlm.nih.gov/Blast.cgi) was used. The functional domains present in the protein were located using the NCBI conserved domain database (http://www.ncbi.nlm.nih.gov/Structure/cdd/wrpsb.cgi).

### Southern blot analysis

Southern blot analyses were carried out as described by Canton et al. [56]. For this purpose, 10 μg of *P. pinaster* genomic DNA was used for each enzymatic digestion. Restriction enzymes used were *BamHI*, *SalI* and *HindIII*. A genomic fragment encoding the carboxy terminus of the protein was used as the probe for hybridization for each *GOGAT* gene Southern blot.

### Sequence alignment and phylogenetic analysis

The sequences used for alignment and phylogenetic trees were obtained from NCBI (http://www.ncbi.nlm.nih.gov/). The accession numbers of the genes used in this study are listed in Additional file 2: Table S1. The CLUSTALW program was used for sequence alignments [57]. The phylogenetic reconstruction was made by the maximum likelihood statistical method, and the phylogeny was tested with 50 bootstrap replications. The evolutionary distances were computed using the JTT matrix-based method [58], and the rates among sites were uniform. The nearest-neighbor -interchange (NNI) was used as the ML heuristic method, and NJ/BioNJ was selected as the initial tree. All these analyses were conducted in MEGA6 [59].

### Protein functional domains

Transit peptides were calculated by comparing the Fd-GOGAT sequences of *Synechocystis sp. PCC 6803* (1LLW) and NADPH-GOGAT sequence of *Azospirillum brasilense* (2VDC) from the Protein Data Bank http://www.rcsb.org/pdb/home/home.do. Positions of the [4Fe-4S] clusters, glutaminase domain, FMN-binding interface, and NAD-binding domains in Fd- and NADH-GOGAT proteins of *P. pinaster* were calculated by comparing the corresponding sites defined for the *P. trichocarpa* protein sequences obtained from the NCBI database (http://www.ncbi.nlm.nih.gov/). The [3Fe–4S] clusters were calculated from the sequences of *Synechocystis sp.* PCC 6803 and *A. brasilense*, according to [25, 34, 47].

### RNA isolation and qPCR

The isolation of RNA from 1-month-old seedlings or 1-year-old plants was performed as described previously [60]. RNase-Free DNase (Promega Corporation, Madison, WI) was used for removal of genomic DNA from the RNA samples. The cDNA synthesiswas performed with iScript Reverse Transcription Supermix (Bio-Rad). Real-time PCR (qPCR) was performed as previously [60]. *Actin* and *elongation factor 1-alpha (EF1α)* were used as reference genes.

## Additional files

Additional file 1: Figure S1. Expression analysis of the pine NADH-GOGAT gene in seedlings (1-month-old), trees (one-year-old) (PDF 62 kb)

Additional file 2: Table S1. Accession numbers of the genes used in this study. (DOC 60 kb)

Additional file 3: Table S2. Exon length of the *Fd-GOGAT* gene from *P. taeda* and the *NADH-GOGAT* gene from *P. pinaster. (DOCX 20 kb)*

Additional file 4: Figure S2. A) *Fd-GOGAT* gene exon/intron structure from different organisms. The number of bp of each gene, the number of exons and the corresponding organisms are indicated. B) *NADH-GOGAT* gene exon/intron structure from different organisms. (PDF 74 kb)

Additional file 5: Table S3. Intron length of the *Fd-GOGAT* gene from *P. taeda* and the *NADH-GOGAT* gene from *P. pinaster. (DOCX 20 kb)*

Additional file 6: Figure S3. Comparison of Fd--‐GOGAT and NAH--‐GOGAT proteins from different organisms. (PDF 72 kb)

## Abbreviations

Fd: Ferredoxin
GOGAT: Glutamate synthase
GS: Glutamine synthetase
IME: Intron-mediated enhancement
LTR: Long terminal repeat
NADH: Nicotinamide adenine dinucleotide
