# Supplementary material for: Glutamate synthases from conifers: gene structure and phylogenetic studies
Source: BMC Genomics. 2018 Jan 19;19:65. doi: 10.1186/s12864-018-4454-y (PMC5775586; doi:10.1186/s12864-018-4454-y)

Supplementary Figure 1: Expression analysis of the pine NADH-GOGAT gene in seedlings (1-month-old), trees (one-year-old)

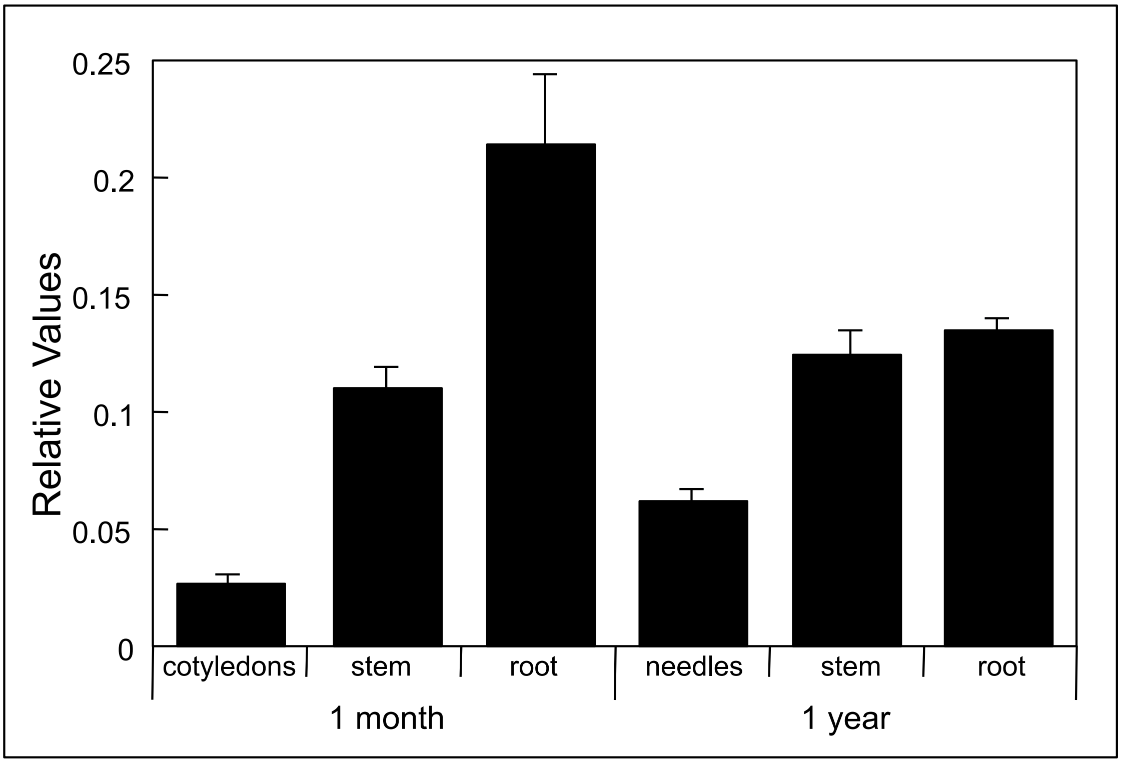

Supplement: Supplementary file 1 — Expression analysis of the pine NADH-GOGAT gene in seedlings (1-month-old), trees (one-year-old) (PDF 62 kb) [file 12864_2018_4454_MOESM1_ESM.pdf]
