# Supplementary material for: Glutamate synthases from conifers: gene structure and phylogenetic studies
Source: BMC Genomics. 2018 Jan 19;19:65. doi: 10.1186/s12864-018-4454-y (PMC5775586; doi:10.1186/s12864-018-4454-y)
Supplement: Supplementary file 2 — Accession numbers of the genes used in this study. (DOC 60 kb) [file 12864_2018_4454_MOESM2_ESM.doc]

Supplementary Table 1: Accession numbers of the genes used in this study

|  | **NCBI** | **NCBI** | **GenBank** | **GenBank** | **Phytozome** |
| --- | --- | --- | --- | --- | --- |
|  | **Fd-GOGAT** | **NADH-GOGAT** | **Fd-GOGAT** | **NADH-GOGAT** | **Fd-GOGAT** |
| *Populus trichocarpa* | XP_002308884.2 | XP_006376642.1 |  |  |  |
| *Ricinus communis* | XP_002526914.1 |  |  | EEF48957.1 |  |
| *Glycine max* | XP_006576787.1 | XP_003553839.1 |  |  |  |
| *Arabidopsis thaliana* |  | NP_001190530.1 | AED90703.1 |  |  |
| *Brachypodium distachyon* | XP_003559858.1 | XP_003566997.1 |  |  |  |
| *Oryza sativa* | XP_015646712.1 | XP_015649242.1 |  |  |  |
| *Klebsormidium* *flaccidum* |  |  | GAQ84369.1 | GAQ85470.1 |  |
| *Physcomitrella patens* | XP_001776900.1 | XP_001767418.1 |  |  |  |
| *Selaginella moellendorffii* | XP_002990959.1 | XP_002965279.1 |  |  |  |
| *Gonium pectorale* |  |  | KXZ47407.1 | KXZ50144.1 |  |
| *Volvox carteri* |  | XP_002958237.1 |  |  | Vocar.0006s0290 |
| *Chlamydomonas reinhardtii* | XP_001703001.1 | XP_001693082.1 |  |  |  |
| *Thalassiosira pseudonana* | XP_002294462.1 | XP_002293590.1 (α)  XP_002291583.1 (β) |  |  |  |
| *Phaeodactylum tricornutum* | XP_002176769.1 | XP_002184279.1 (α)  XP_002180120.1 (β) |  |  |  |
| *Guillardia theta* | XP_005828144.1 | XP_005822697.1 |  |  |  |
| *Bangia fuscopurpurea* | AKE98974.1 |  |  |  |  |
| *Porphyra purpurea* |  |  | AAC08261.1 |  |  |
| *Monosiga brevicollis* |  | XP_001743961.1 |  |  |  |
| *Laccaria bicolor* |  | XP_001878616.1 |  |  |  |
| *Candida tropicalis* |  | XP_002549937.1 |  |  |  |
| *Penicillium rubens* |  | XP_002565078.1 |  |  |  |
| *Crassostrea gigas* |  | XP_011440994.1 |  |  |  |
| *Opisthorchis viverrini* |  | XP_009165784.1 |  |  |  |
| *Branchiostoma floridae* |  | XP_002612218.1 |  |  |  |
| *Bombyx mori* |  | NP_001041678.1 |  |  |  |
| *Cyanothece sp. PCC 7424* | WP_012598855.1 | WP_015955630.1 (α) WP_015955632.1 (β) |  |  |  |
| *Pleurocapsa sp. PCC 7327* | WP_015145655.1 | WP_015146031.1 (α)  WP_015146028.1 (β) |  |  |  |
| *Synechocystis sp. PCC 6803* |  |  | BAA18693.1 | BAA17018.1 (α)  BAA16777.1 (β) |  |
| *Stanieria cyanosphaera* |  |  | AFZ35834.1 | AFZ35796.1 (α)  AFZ35797.1 (β) |  |
| *Azospirillum brasilense* |  |  |  | AAA22179.1 (α)  AAG38999.1 (β) |  |
