# Supplementary material for: Glutamate synthases from conifers: gene structure and phylogenetic studies
Source: BMC Genomics. 2018 Jan 19;19:65. doi: 10.1186/s12864-018-4454-y (PMC5775586; doi:10.1186/s12864-018-4454-y)
Supplement: Supplementary file 3 — Exon length of the Fd-GOGAT gene from P. taeda and the NADH-GOGAT gene from P. pinaster. (DOCX 20 kb) [file 12864_2018_4454_MOESM3_ESM.docx]

| Exon | *Fd-GOGAT*  Length (bp) | *NADH-GOGAT*  Length (bp) |
| --- | --- | --- |
| **E**1 | 300 | 426 |
| **E**2 | 313 | 120 |
| **E**3 | 444 | 105 |
| **E**4 | 131 | 153 |
| **E**5 | 100 | 101 |
| **E**6 | 70 | 97 |
| **E**7 | 100 | 110 |
| **E**8 | 92 | 104 |
| **E**9 | 145 | 180 |
| **E**10 | 161 | 344 |
| **E**11 | 112 | 71 |
| **E**12 | 214 | 470 |
| **E**13 | 157 | 873 |
| **E**14 | 131 | 155 |
| **E**15 | 58 | 162 |
| **E**16 | 106 | 1599 |
| **E**17 | 99 | 219 |
| **E**18 | 116 | 269 |
| **E**19 | 197 | 306 |
| **E**20 | 114 | 268 |
| **E**21 | 238 | 258 |
| **E**22 | 97 | 267 |
| **E**23 | 96 |  |
| **E**24 | 198 |  |
| E25 | 77 |  |
| E26 | 127 |  |
| **E**27 | 99 |  |
| **E**28 | 143 |  |
| **E**29 | 95 |  |
| **E**30 | 238 |  |
| **E**31 | 81 |  |
| **E**32 | 83 |  |
| **E**33 | 161 |  |

**Supplementary Table 2.** Exon length of the *Fd-GOGAT* gene from *P. taeda* and the *NADH-GOGAT* gene from *P. pinaster*
