# Supplementary material for: Glutamate synthases from conifers: gene structure and phylogenetic studies
Source: BMC Genomics. 2018 Jan 19;19:65. doi: 10.1186/s12864-018-4454-y (PMC5775586; doi:10.1186/s12864-018-4454-y)

Supplementary Figure 2: A) *Fd-GOGAT* gene exon/intron structure from different organisms. The number of bp of each gene, the number of exons and the corresponding organisms are indicated. B) *NADH-GOGAT* gene exon/intron structure from different organisms. The number of bp of each gene, the number of exons and the corresponding organisms are indicated. In both diagrams, exons are in orange and introns in black.

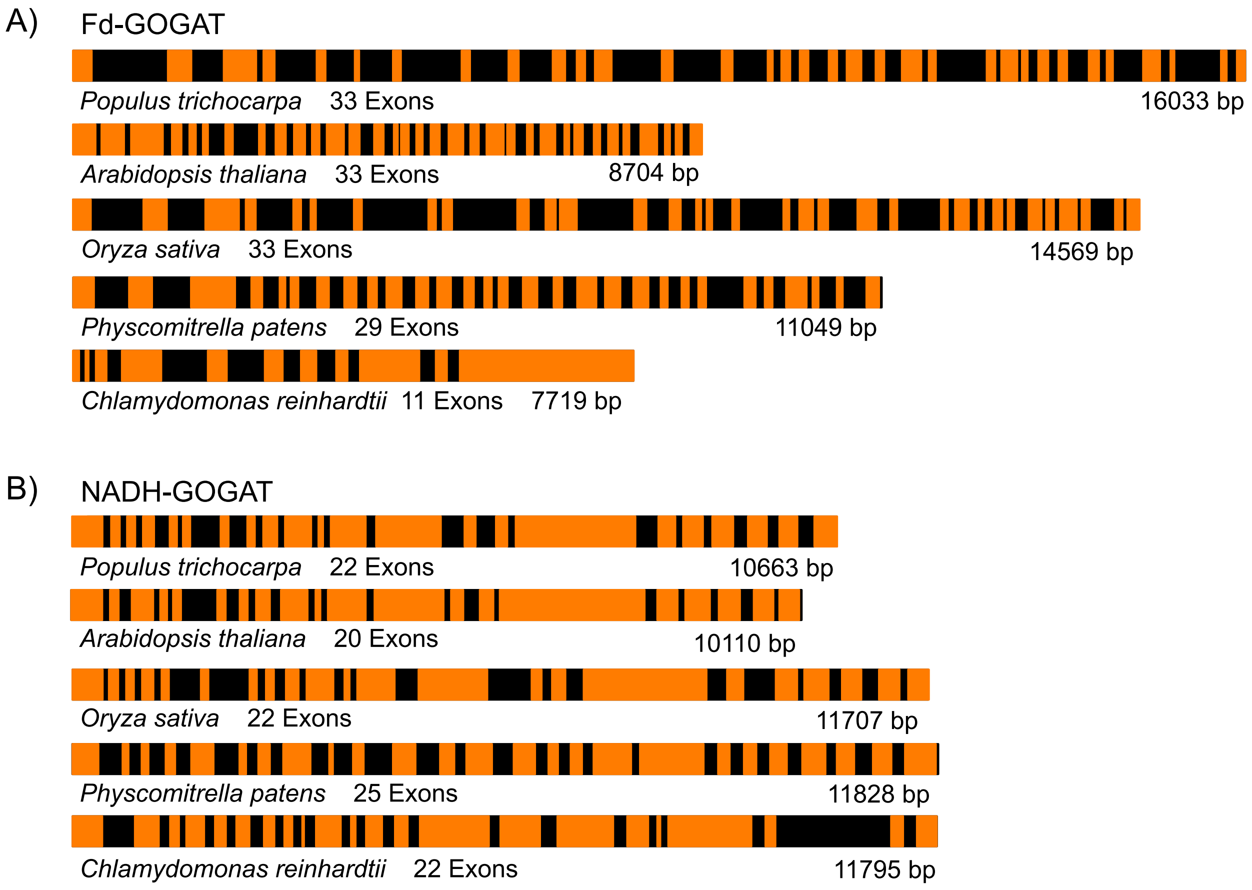

Supplement: Supplementary file 4 — A) Fd-GOGAT gene exon/intron structure from different organisms. The number of bp of each gene, the number of exons and the corresponding organisms are indicated. B) NADH-GOGAT gene exon/intron structure from different organisms. (PDF 74 kb) [file 12864_2018_4454_MOESM4_ESM.pdf]
