# Supplementary material for: Glutamate synthases from conifers: gene structure and phylogenetic studies
Source: BMC Genomics. 2018 Jan 19;19:65. doi: 10.1186/s12864-018-4454-y (PMC5775586; doi:10.1186/s12864-018-4454-y)
Supplement: Supplementary file 5 — Intron length of the Fd-GOGAT gene from P. taeda and the NADH-GOGAT gene from P. pinaster. (DOCX 20 kb) [file 12864_2018_4454_MOESM5_ESM.docx]

| Intron | *Fd-GOGAT*  length  (bp) | *NADH-GOGAT*  length  (bp) |
| --- | --- | --- |
|  |  |  |
| **I**1 | 52136 | 276 |
| **I**2 | 1352 | 1103 |
| **I**3 | 195 | 745 |
| **I**4 | 346 | 184 |
| **I**5 | 511 | 743 |
| **I**6 | 21322 | 569 |
| **I**7 | 269 | 117 |
| **I**8 | 33655 | 83 |
| **I**9 | 22232 | 469 |
| **I**10 | 27971 | 86 |
| **I**11 | 164 | 97 |
| **I**12 | 31450 | 165 |
| **I**13 | 1095 | 188 |
| **I**14 | 7847 | 232 |
| **I**15 | 222 | 461 |
| **I**16 | 31870 | 196 |
| **I**17 | 43254 | 232 |
| **I**18 | 187 | 122 |
| **I**19 | 1315 | 95 |
| **I**20 | 399 | 112 |
| **I**21 | 251 | 323 |
| **I**22 | 19028 |  |
| **I**23 | 134 |  |
| **I**24 | 95 |  |
| **I**25 | 662 |  |
| **I**26 | 120 |  |
| **I**27 | 13642 |  |
| **I**28 | 137 |  |
| **I**29 | 85 |  |
| **I**30 | 99 |  |
| **I**31 | 13056 |  |
| **I**32 | 245 |  |
|  |  |  |

**Supplementary Table 3.** Intron length of the *Fd-GOGAT* gene from *P. taeda* and the *NADH-GOGAT* gene from *P. pinaster*
