# Supplementary material for: Glutamate synthases from conifers: gene structure and phylogenetic studies
Source: BMC Genomics. 2018 Jan 19;19:65. doi: 10.1186/s12864-018-4454-y (PMC5775586; doi:10.1186/s12864-018-4454-y)

Supplementary Figure 3: Comparison of Fd-GOGAT and NAH-GOGAT proteins from different organisms. The sizes of the proteins are proportionally represented. The number of amino acids of each polypeptide is indicated in brackets

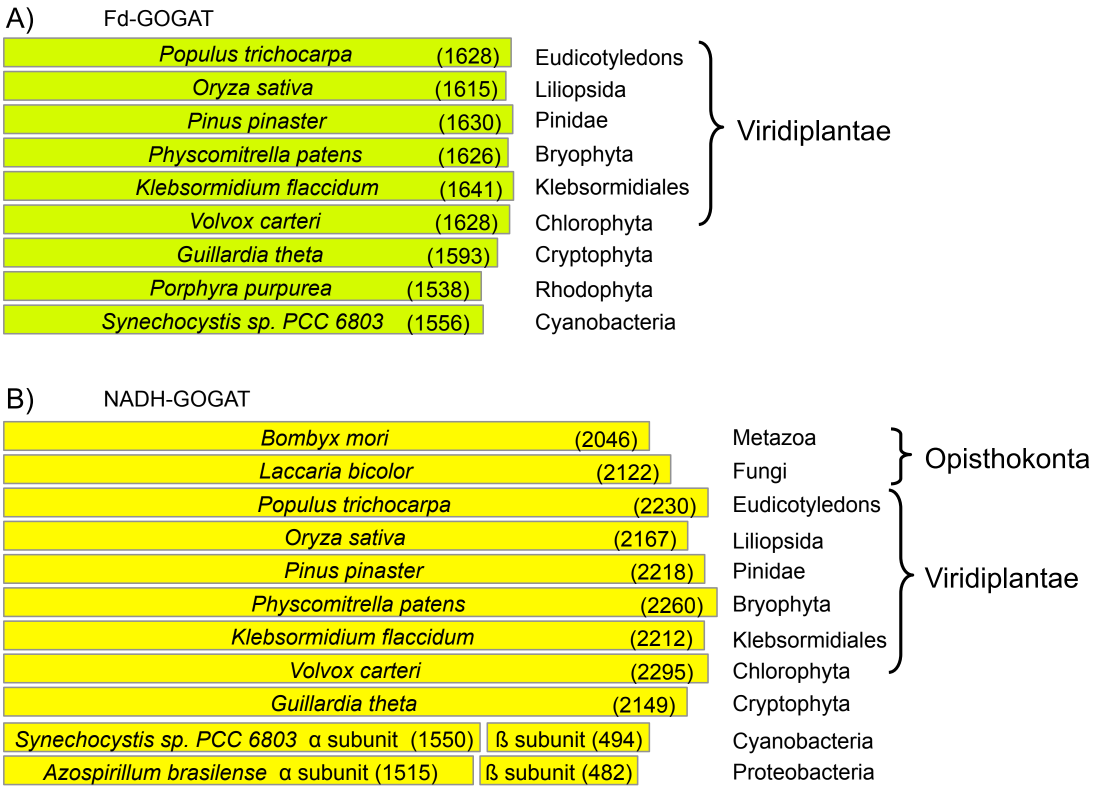

Supplement: Supplementary file 6 — Comparison of Fd--‐GOGAT and NAH--‐GOGAT proteins from different organisms. (PDF 72 kb) [file 12864_2018_4454_MOESM6_ESM.pdf]
